# Portuguese version of the COVID-19 perceived risk scale – psychometric study

**DOI:** 10.1192/j.eurpsy.2021.825

**Published:** 2021-08-13

**Authors:** A.T. Pereira, C. Cabaços, P. Paredes, T. Soares, A. Araujo, R. Sousa, A. Macedo

**Affiliations:** 1 Institute Of Psychological Medicine, Faculty of Medicine, University of Coimbra, Coimbra, Portugal; 2 Institute Of Psychological Medicine, Faculty Of Medicine, University of Coimbra, coimbra, Portugal; 3 Usf Coimbra Centro, USF Coimbra Centro, Coimbra, Portugal; 4 Institute Of Psychological Medicine, Faculty Of Medicine, University of Coimbra, Coimbra, Portugal

**Keywords:** CFA, COVID-19, Perceived Risk Scale, EFA

## Abstract

**Introduction:**

Risk perception of COVID-19 is potentially a significant determinant of the pandemic evolution and the public’s response to it. Acceptable levels of risk perception can be considered good for people to effectively fight the pandemic and adopt preventive health behaviors while high levels of risk perception may be damaging. Recently, Yıldırım&Güler (2020) developed the Covid-19 Perceived Risk Scale (C19PRS) to measure this construct.

**Objectives:**

To analyze the psychometric properties of the C19PRS Portuguese version, namely construct validity, internal consistency and convergent validity.

**Methods:**

A community sample of 234 adults (75.6% women; mean age= 29.53±12.51; range:16-71) completed an on-line survey with the Portuguese versions of the CPRS and the Fear of Covid-19 Scale (FCV-19S; Cabaços et al. 2020). The total sample was randomly divided in two sub-samples: sample A (n=117) was used to perform an exploratory factor analysis/EFA; sample B (n=117) to make a confirmatory factor analysis/CFA.

**Results:**

EFA resulted in three components. CFA revealed that the second-order model with three factors presented good fit indexes (X2/df=1.471; CFI=.959; GFI=.948; TLI=.932; p[RMSEA≤.01]=.065). CPRS Cronbach alphas was α=.687; for F1 Worry, F2 Susceptibility to Covid-19 and F3 Susceptibility to Overall Morbimortality were α=.747, α=.813 and α=.543, respectively. The total and dimensional scores significantly correlated with FCV-19S (r>.30, p<.01).

**Conclusions:**

This study provides evidence for the validity and reliability of the Portuguese version of CPRS, which will be used in an ongoing research project on the relationship between Covid-19 perceived risk, perfectionism, cognitive processes and adherence to public health measures to contain the pandemic.

